# How to Choose the Optimal Starting Dose of Clomiphene Citrate (50 or 100 mg per Day) for a First Cycle of Ovulation Induction in Anovulatory PCOS Women?

**DOI:** 10.3390/jcm12154943

**Published:** 2023-07-27

**Authors:** Lucie Huyghe, Camille Robin, Agathe Dumont, Christine Decanter, Maeva Kyheng, Didier Dewailly, Sophie Catteau-Jonard, Geoffroy Robin

**Affiliations:** 1 Department of Reproductive Medicine and Fertility Preservation, Lille University Hospital, 59000 Lille, France; doclhuyghe@gmail.com (L.H.); camille.robin@chu-lille.fr (C.R.); agathe.dumont@hotmail.fr (A.D.); sophie.jonard@chu-lille.fr (S.C.-J.); 2 Department of Biostatistics, Lille University Hospital, 59000 Lille, France; 3 ULR 2694—METRICS: Evaluation des Technologies de Santé et des Pratiques Médicales, University of Lille, 59000 Lille, France; 4 Faculty of Medicine, University of Lille, 59000 Lille, France; 5 UMRS-1172, Laboratory of Development and Plasticity of the Neuroendocrine Brain, Jean-Pierre Aubert Research Centre, 59000 Lille, France; 6 Department of Medical Gynecology and Sexology, Lille University Hospital, 59000 Lille, France

**Keywords:** polycystic ovary syndrome, clomiphene citrate, ovulation induction, antimullerian hormone, sex hormone binding globulin

## Abstract

Research question: Clomiphene citrate (CC) is one of the first-line treatments for ovulation induction in women with anovulatory polycystic ovary syndrome (PCOS). However, nearly 1 out of 2 women is resistant to 50 mg/day of CC. The objective of this study is to investigate the clinical, biological, and/or ultrasound factors that may predict the resistance to 50 mg/day of CC in the first cycle of treatment in women with anovulatory PCOS. This would make it possible to identify PCOS patients to whom the dose of 100 mg/day would be offered as of the first cycle. Design: A retrospective and monocentric study was conducted on 283 women with anovulatory PCOS who required the use of ovulation induction with CC (903 cycles). Results: During the first cycle of treatment, 104 patients (36.8%) were resistant to 50 mg/day of CC. Univariate regression analysis showed that patients who resisted 50 mg/day of CC had significantly higher BMI, waist circumference, serum levels of AMH, total testosterone, Δ4-androstenedione, 17-OHP, and insulin (*p* < 0.05), compared to patients ovulating with this dose. Serum levels of SHBG were significantly lower in patients resistant to 50 mg/day (*p* < 0.05). After multivariate analysis, only AMH and SHBG remained statistically significant (*p* = 0.01 and *p* = 0.001, respectively). However, areas under the ROC curves were weak (0.59 and 0.68, respectively). Conclusion: AMH and SHBG are the only two parameters significantly associated with the risk of resistance to 50 mg/day of CC. However, no satisfactory thresholds have been established to predict resistance to 50 mg CC.

## 1. Introduction

Polycystic ovary syndrome (PCOS) is the most common endocrine disorder in women of childbearing age, with a prevalence ranging from 4% to 21%, depending on the diagnostic criteria used [1]. The presentation of this syndrome is very heterogeneous, with variable clinical expression, including usually menstrual cycle disorders, hyperandrogenism, and/or infertility [2]. Since 2004, the ESHRE/ASRM Rotterdam Consensus criteria are the most commonly used for the diagnosis of PCOS [3].

PCOS is the mean etiology of infertility due to anovulation [2,4,5]. Clomiphene citrate (CC) and letrozole are the first-line treatment for ovulation induction in PCOS [4,5,6]. Both CC and letrozole are successful in inducing pregnancy. A recent meta-analysis confirms a moderate but significant superiority of letrozole over clomiphene citrate [7]. Thus, in countries where letrozole is off-label for ovulation induction, such as France, CC is used as a first-line treatment for ovulation induction in PCOS women [6].

CC is a selective modulator of estrogen receptors exerting anti-estrogenic activity at the hypothalamic level. CC enhances the pulsatile release of GnRH. This will result in an increase in the secretion of endogenous gonadotropins and especially follicle-stimulating hormone (FSH) by the anterior pituitary gland, allowing cyclic follicular growth [2,8,9,10]. The recommended starting dose of CC is 50 mg/day for 5 days. If ovulation is not achieved, the dose will then be increased by 50 mg/day for 5 days in the next cycle, up to a maximum dose of 150 mg/day for 5 days [11,12]. As reported by some authors, the ovulation rate with this treatment can be as high as 75–80% [10,11]. Despite the relatively high efficacy of CC, approximately 15–40% of patients will not respond to the maximum recommended dose of 150 mg/day for 5 days and will be considered resistant to this treatment [13,14,15,16].

Several studies have investigated factors that may potentially predict CC resistance, comparing treatment-sensitive patients with those resistant to CC 150 mg/day. Thus, obesity, insulin resistance, hyperandrogenism, and excess AMH are among the most common factors associated with CC resistance [4,17,18,19]. Genetic predisposition would also play a role in CC resistance [20]. However, all these studies failed to identify clinical, hormonal, metabolic, or ultrasound factors which could predict with certainty a complete resistance to CC. Thus, in countries where clomiphene citrate is the only first-line treatment available for ovulation induction in PCOS women, there is, therefore, no factor that would make it possible to immediately opt for second-line treatments (ovarian drilling or ovulation induction with gonadotropins) [4,5,6]. Therefore, it would be interesting to investigate clinical, biological, and/or ultrasound factors that may predict resistance to the initial CC dose of 50 mg/day. Indeed, nearly 1 in 2 women is resistant to 50 mg/day of CC [18,21]. Identifying this or these factor(s) would allow starting the treatment at a higher dose (100 mg/day) at once. This would save significant time for patients who are resistant to 50 mg/day of CC. Moreover, the fact of being able to achieve pregnancy more quickly also theoretically limits the risk of having to use second-line treatments: either injectable gonadotropins or laparoscopic ovarian drilling [4,5,6]. These two treatments are both more expensive and more prone to complications (e.g., higher risk of multiple pregnancies with injectable gonadotropins, requiring rigorous ultrasound and hormonal monitoring, or the operative and anesthetic risks of ovarian drilling) rather than first-line ovulation inducers such as clomiphene citrate [5,22]. To our knowledge, no study has investigated this issue.

The main objective of our study was, therefore, to investigate the clinical, biological, and/or ultrasound parameters which would predict resistance to CC at 50 mg/day in women with anovulatory PCOS. The secondary objective was to determine the effectiveness of anovulation management with this ovulation inducer in the whole cohort by trying to determine the optimal number of initiated cycles and ovulatory cycles to offer to these PCOS women.

## 2. Materials and Methods

### 2.1. Population

This is a retrospective, single-center study conducted between May 2003 and December 2020 in the Reproductive Medicine Department of Lille University Hospital in France. As this study was retrospective and without intervention, the opinion of the Ethics Committee on the study was not required. All patients had given prior consent for the use of their clinical, hormonal, and ultrasound records. On 16 December 2019, the Institutional Review Board of the Lille University Hospital gave unrestricted approval for the anonymous use of all patients’ clinical, hormonal, and ultrasound records (reference DEC20150715-0002).

All anovulatory PCOS women treated with a starting dose of CC at 50 mg/day were included in the study. The diagnosis of PCOS was based on the Rotterdam criteria published in 2003 [3]. Two of the following three criteria had to be present for diagnosis: (1) Oligo- or anovulation (OA): oligomenorrhea (<8 cycles per year), amenorrhea (absence of menses > 3 months) or regular anovulatory cycles (menstrual cycles between 26 and 34 days but with no progesterone increase above 3 ng/mL 7 to 8 days before menstruation [23]); all the women in our study suffer from OA. (2) Clinical hyperandrogenism (HA) (modified Ferriman and Gallwey score ≥ 7 in our Caucasian population [24] or biological hyperandrogenism (total testosteronemia ≥ 0.50 ng/mL, as previously described [25,26]). (3) Ultrasound polycystic ovaries (PCOM): ovarian volume ≥ 10 mL [27,28] and/or ovarian surface area ≥ 5.5 cm^2^ [25], and/or FNPO (follicle number per ovary) ≥ 12 from 2003 to 2007 [3,27] (ultrasound scanner: General Electric Logic 400, Milwaukee, equipped with a 7 MHz endovaginal probe), then FNPO ≥ 19 from 2008 (ultrasound scanner: General Electric Voluson E8, equipped with a 5 to 9 MHz endovaginal probe) [26]. The presence of elevated AMH was considered equivalent to the presence of polycystic ovaries on ultrasound (serum AMH ≥ 35 pmol/L), as previously reported [26,29,30]. The PCOS phenotype was then identified for each patient, according to the NIH 2012 extension of the Rotterdam classification [1], as described above [30]: phenotype A (OA + HA + PCOM), phenotype B (OA + HA), phenotype D (OA + PCOM). Given our inclusion criteria, phenotype C (HA + PCOM = ovulatory PCOS) is not present in our population.

The exclusion criteria were women aged under 18 years or over 43 years, other etiologies of hyperandrogenism or dysovulation (hyperprolactinemia, nonclassical adrenal hyperplasia, organic or functional gonadotropic deficiencies, ovarian or adrenal tumors, Cushing’s syndrome, thyroid dysfunctions, idiopathic dysovulation, and premature ovarian failure), endometriosis, alterations in tubal permeability, sperm abnormalities. We also excluded PCOS women with metformin treatment.

Clinical examination, hormonal and metabolic tests, and pelvic ultrasound examination were performed between the second and the fifth days of the menstrual cycle, either spontaneous or after a progestin challenge test (dydrogesterone, 10 mg/day for 7 to 10 days).

The clinical examination included a detailed interview, seeking, in particular, to specify the duration of menstrual cycles (regular cycles, oligomenorrhea, or amenorrhea), BMI calculation, waist circumference measurement, and assessment of hirsutism according to the modified Ferriman and Gallwey score [24]. The biological assessment carried out at the beginning of the follicular phase, between the second and the fifth days of the menstrual cycle, included measurements of estradiol, LH and FSH, AMH, total testosterone, Δ4-androstenedione, 17-hydroxy-progesteron, SDHEA, SHBG, TSH, prolactin, and insulin. As previously described [31,32,33,34], estradiol, LH, FSH, total testosterone, Δ4-androstenedione, 17-hydroxy-progesterone, SDHEA, SHBG, and prolactin were measured by immunoassays. Until January 2016, the AMH assay was performed using the second-generation AMH-EIA enzyme immunoassay kit from Beckman Coulter Immunotech (manual technique) as previously described [26,30]. From January 2016, AMH was measured using an automated method, Access Dxi, marketed by Beckman Coulter. We chose to perform the statistical analyses considering the values obtained with the AMH-EIA test. The conversion formula was applied for all AMH values obtained with the Access Dxi test, i.e., for all assays performed after January 2016: AMH-EIA = (AMH Dxi − 0.44)/0.775 (values expressed in pmol/L), as previously published [35]. The pelvic ultrasound was performed on the same day. In addition to the search for uterine or tubal pathologies, a count of antral follicles (follicles strictly less than 10 mm in diameter) was conducted during this examination. Antral follicular count (CFA) and follicle number per ovary (FNPO) was performed using “the Real-time 2D ultrasound” method [36], and ovarian surfaces using a manual ellipse, as described previously [25]. From 2002 to 2008, the ultrasound machine used was a General Electric Milwaukee Logic 400, with a 7 MHz endovaginal probe; then, from 2008, a General Electric Voluson 28, with a 5 to 9 MHz endovaginal probe. A complete infertility work-up was performed on both members of a couple before considering a CC ovulation induction treatment. Bilateral tubal patency was checked in the patient by hysterosalpingography or laparoscopy. The patient’s partner had to perform at least one spermogram to ensure compatibility with spontaneous pregnancy.

### 2.2. Therapeutic

All patients were treated with simple CC ovulation induction therapy. In the first cycle, the initial dose was 50 mg/day, starting on the second day of a spontaneous cycle or triggered by sequential treatment with dydrogesterone, 10 mg/day for 7 to 10 days.

The ovarian response was systematically monitored by ovulation ultrasound monitoring, starting around D12 (±1 day), in the Reproductive Medicine Department of Lille University Hospital (France). No biological assay was performed during follicular growth monitoring.

The purpose of the ultrasound was to evaluate the response to CC by measuring the number of selected follicles (≥10 mm) and to measure the endometrial thickness in order to identify a possible anti-estrogenic effect of CC on the endometrium [37].

The date of ovulation was estimated from the size of the dominant follicle measured on ultrasound. A progesterone assay was performed approximately 7–8 days after the estimated date of spontaneous ovulation. A significant increase in progesterone levels confirms spontaneous ovulation and, thus, a positive response to CC.

### 2.3. Cycle Outcome

In the absence of menstruation approximately 2 weeks after the estimated date of ovulation, a plasma hCG test was performed. If the blood pregnancy test was positive, a pelvic ultrasound was performed at about 6 weeks of amenorrhea to ensure that the pregnancy was ongoing and screen for multiple pregnancies. In the absence of pregnancy, however, the patient would start a new cycle of CC at the dose at which ovulation was achieved.

In case of the absence of follicular recruitment (confirmed by another ultrasound examination performed 5 to 7 days after the first), sequential treatment with 10 mg/day of dydrogesterone for 7 to 10 days was prescribed to induce menstruation. The dosage of CC was then increased to the next cycle in 50 mg increments, with a maximum dose of 150 mg/day. A maximum of 6 ovulatory cycles with CC was performed for achieving a clinical pregnancy.

### 2.4. Response to CC

The response to 50 mg CC was assessed by the presence or absence of ovulation via ultrasound monitoring and serum progesterone assay. The patient was considered sensitive to CC if the progesterone level in the second half of the cycle was ≥3 ng/mL. In the absence of follicular recruitment, the patient was then considered resistant to 50 mg CC.

Excessive responsiveness to CC was defined by the presence of three or more dominant follicles on ultrasound monitoring.

We have also evaluated the effectiveness of the current strategy of gradually increasing CC doses in the event of resistance to treatment by calculating the cumulative rates of progressive pregnancies per cycle initiated and per CC ovulatory cycle.

### 2.5. Statistical Analysis

Baseline characteristics were compared between the two groups using Chi-square tests (or Fisher’s exact tests when expected cell frequency was <5) for categorical characteristics and the Student *t*-test (or Mann−Whitney U test for non-Gaussian distribution) for continuous characteristics.

To assess the independent predictors of the 50 mg resistance, baseline characteristics associated with a *p* < 0.20 in univariate analyses were implemented into a backward-stepwise multivariable logistic regression model using a removal criterion of *p* > 0.05. Results were expressed using Odds ratios (ORs) as effect sizes with 50 mg non-resistance as reference. Before developing the multivariable models, we examined the log-linearity assumption for continuous characteristics using restricted cubic spline functions [38], as well as the absence of colinearity between candidate predictors by calculating the variance inflation factors (VIFs). Because of the similarity and collinearity between SHBG and insulinemia, obesity and BMI, we decided to perform the multivariate model with SHBG and BMI. To avoid case deletion due to missing data on baseline characteristics and outcomes, missing data were imputed by multiple imputations using a regression-switching approach [39] (chained equations with m = 10) also before developing the multivariable model [39]. The imputation procedure was performed under the missing at random assumption using all baseline characteristics and study outcomes with a predictive mean matching method for continuous variables and logistic regression models (binary, ordinal or multinomial) for categorical variables. Estimates obtained in the different imputed data sets were combined using Rubin’s rules [40].

Finally, we determined the optimal threshold value of factors associated with 50 mg resistance in the final models by maximizing the Youden index from the ROC curves. Statistical testing was conducted at the two-tailed α-level of 0.05. Data were analyzed using the SAS software version 9.4 (SAS Institute, Cary, NC, USA).

## 3. Results

A total of 283 patients with anovulatory PCOS were included in the study between May 2003 and December 2019, representing a total of 903 cycles of CC. The clinical, biological, and ultrasound characteristics of our population are detailed in Table 1.

**Figure 1:**
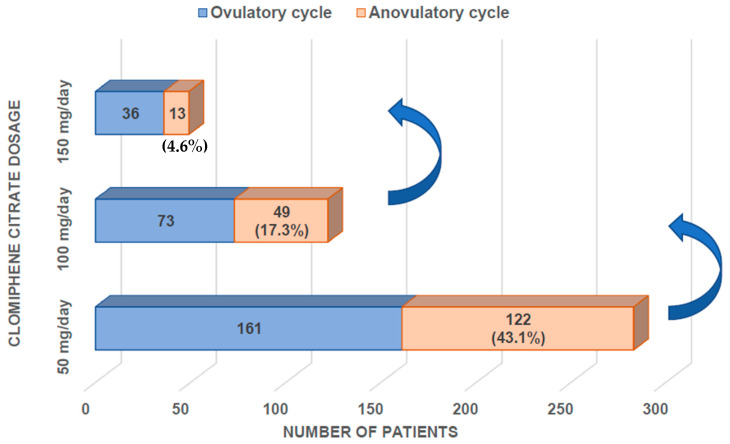
Distribution of anovulatory PCOS women who do or do not ovulate after CC induction of ovulation in incremental daily doses of 50, 100, or 150 mg for 5 subsequent days.

Figure 1 illustrates the distribution of anovulatory PCOS women who do or do not ovulate after CC induction of ovulation in incremental daily doses of 50, 100, or 150 mg for 5 subsequent days. A total of 161 patients (56.9%) ovulated during the first cycle of CC treatment at 50 mg/day. Therefore, 122 patients (43.1%) were resistant to 50 mg/day of CC during the first cycle of treatment. Otherwise, 24 patients (8.5%) were hyperresponsive during the first cycle of CC at 50 mg/day. Finally, 49 patients (17.3%) were also resistant to the dose of 100 mg/day, and 13 patients (4.6%) were resistant to the dose of 150 mg/day.

**Figure 2:**
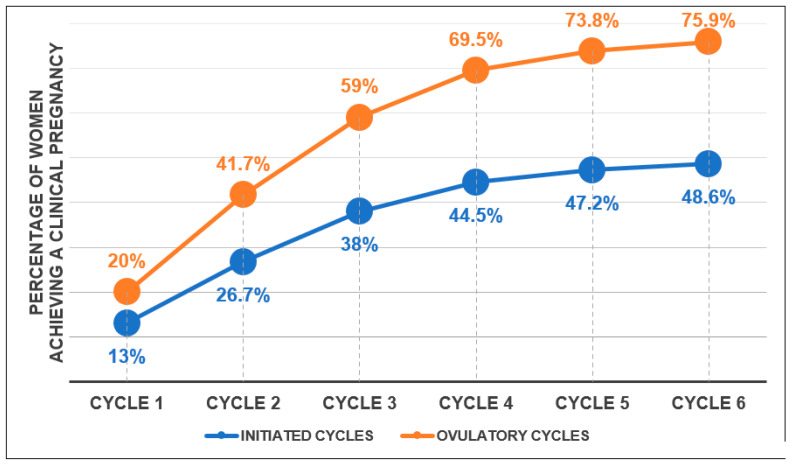
Cumulative rates of clinical pregnancy, per initiated and ovulatory CC cycle, at 50, 100, and 150 mg CC during 5 subsequent days.

Figure 2 shows the cumulative clinical pregnancy rates per CC-initiated cycle and per CC ovulatory cycle at 50, 100, and 150 mg per day.

To investigate predictors of resistance to 50 mg CC in the first cycle of treatment, univariate regression analysis was performed to compare the clinical, biological, and ultrasound characteristics of patients sensitive to 50 mg CC versus those resistant to this dose. The results of the univariate regression analysis are presented in Table 2. Compared to patients ovulating at the 50 mg dose of CC in the first cycle, patients who were resistant to this same dose had significantly higher BMI, waist circumference, serum levels of AMH, total testosterone, Δ4-androstenedione, 17-OHP, and insulin. In contrast, serum levels of SHBG were significantly lower in patients resistant to 50 mg/day.

Table 3 shows the final model from the multivariate analysis. After multivariate analysis, higher levels of AMH and lower levels of SHBG were statistically associated with a higher risk of resistance at the dose of 50 mg/day of CC (OR = 1.08, 95% CI 1.03 to 1.14) and OR = 0.96, 95% CI 0.94 to 0.99, respectively).

**Figure 3:**
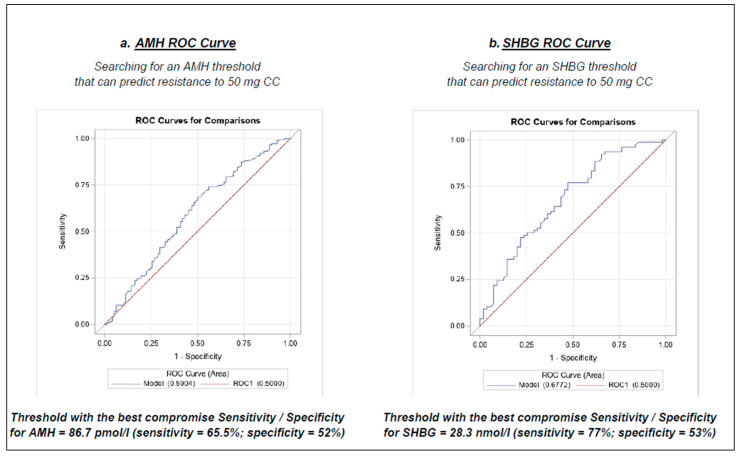
ROC analyses of AMH and SHBG and resistance to 50 mg CC.

We, therefore, sought to establish thresholds for AMH and SHBG that would potentially predict resistance to the 50 mg CC dose in the first cycle of treatment. ROC curves were produced for this purpose. These ROC curves are shown in Figure 3.

Finally, we estimated expected ovulation rates in the first cycle of ovulation induction by CC at the 50 mg/day dose according to the AMH thresholds, the SHBG thresholds, and the AMH and SHBG thresholds when these dosages are combined. The results are presented in Table 4.

## 4. Discussion

CC is an effective ovulation-inducing treatment for women with anovulatory PCOS, as highlighted by the good cumulative clinical pregnancy rates per ovulatory cycle in our study. However, in view of the stagnation of clinical pregnancy rates between rank 4 and rank 6, it seems wise not to continue this treatment beyond 4 consecutive ovulatory cycles. In addition, about a third of women will not respond to the 50 mg dose of CC. Thus, it could be of interest to identify women who present risk factors for resistance to the 50 mg dose of CC to accelerate the onset of clinical pregnancy. Indeed, it would then be relevant to immediately offer a dose of 100 mg/day of CC to these PCOS patients from the first cycle of ovulation induction.

Our study shows that AMH and SHBG appear to be predictive factors for resistance to 50 mg CC during the first cycle of ovulation induction in patients with anovulatory PCOS. Nevertheless, we failed to identify thresholds for both AMH and SHBG, which could be used to predict resistance to 50 mg CC, and thus, to initiate treatment at 100 mg/day.

To our knowledge, this is the first study to investigate predictive factors of resistance to 50 mg CC in the first cycle of ovulation induction in PCOS women. However, many authors have focused on predictors of resistance to 150 mg/day CC. Among these factors, AMH is a parameter frequently cited as a predictor of response to CC. Xi et al. [21] and Mahran et al. [41] consider AMH a good marker of CC response. According to Xi et al. [21], patients with a high AMH level, particularly above 55.5 pmol/L, have a significantly reduced chance of ovulation under CC. According to Mahran et al. [41], the AMH level may help determine the initial CC dose: the higher the AMH level, the greater the initial CC dose should be, with a proposed threshold of 24.3 pmol/L.

As AMH is produced in greater quantities in women with PCOS, its inhibitory action on FSH is therefore more pronounced [32,34,42,43,44]. This may explain the results of our study, which indicate that as serum AMH levels increase, the patient is more likely to be resistant to the initial 50 mg CC dose. The higher the AMH, the greater the doses of FSH required to achieve follicular growth and ovulation. This is in agreement with the results of Köninger et al. [45], suggesting that in CC-resistant women receiving recombinant FSH ovulation stimulation, the higher the patient’s serum AMH level, the greater the doses of FSH required to achieve ovulation. Unfortunately, it was not possible to establish an AMH threshold predictive of resistance at the initial 50 mg CC dose. The selected AMH cut-off was 86.7 pmol/L, but sensitivity and specificity were not satisfactory for clinical application (65.5% and 52%, respectively). The AMH thresholds proposed in the literature to predict total resistance to CC (150 mg/day dose) are highly variable (from 24.3 pmol/L to 88.4 pmol/L) with no satisfactory relative sensitivities and specificities [21,41,46,47,48]. In addition, some authors found no significant difference in serum AMH levels between women ovulating on CC versus those resistant to treatment [49]. The latter explained the difference between these results and those of other teams by the difference in the AMH assay kits used in the different studies, which have different sensitivities. Indeed, there are several AMH test kits around the world, which makes the use of this test difficult to generalize [35,43].

A recent retrospective study has shown that in phenotype B of PCOS, ovarian volume did not have any predictive value of the dosage of CC required to induce ovulation [50]. This study is very interesting, but our population contains very few PCOS with phenotype B. In fact, as we have published previously, using AMH as a biological equivalent of PCOM, there are very few women with phenotype B of PCOS in our population [30]. Finally, to our knowledge, there are no studies demonstrating a clear statistical correlation between ovarian volume and serum AMH levels.

Furthermore, SHBG also appears to be a statistically significant parameter in the multivariate analysis of our study. The lower the SHBG, the greater the risk of resisting the dose of 50 mg/day of CC. Patients with confounding factors that may induce a decrease in SHBG, such as hypothyroidism, were excluded from our study. However, as with AMH, it was not possible to establish a satisfactory threshold for SHBG that would allow us to specify a starting dose of 100 mg CC. The selected cut-off of 28.3 nmol/L (sensitivity of 77% and specificity of 53%) is not useful in clinical practice. SHBG is a plasma transport glycoprotein produced by liver cells, whose role is to regulate the bioavailability of sex steroid hormones [51]. Abnormally low levels of SHBG are frequently observed in women with PCOS (especially in women with android obesity) and contribute to the symptoms of clinical hyperandrogenism observed in these patients by increasing the bioavailable (and therefore bioactive) fraction of circulating androgens (hirsutism, acne, androgenic alopecia) [52,53]. A low serum level of SHBG is considered a marker of insulin resistance (inhibition of hepatic synthesis of SHBG due to compensatory hyperinsulinism). A recent meta-analysis highlights the correlation between SHBG and metabolic dysregulation in PCOS women [54]. According to this meta-analysis, women with PCOS with low levels of SHBG were more likely to suffer from hyperandrogenism, insulin resistance, carbohydrate intolerance, type 2 diabetes, obesity, and cardiovascular disease. Therefore, the data in the literature, as well as the results of our study, demonstrate that a complete metabolic assessment and management of obesity are crucial before treatment in women with PCOS, both for the success of treatment and for the prevention of long-term complications. A recent prospective study indicates that women with more disturbed metabolic parameters were at greater risk of resistance to clomiphene citrate, even when the dose was increased to 150 mg/day for 5 consecutive days [55]. The meta-analysis by Deswal et al. [54] indicates that insulin-sensitizing agents, such as myo-inositol or metformin, can significantly improve the levels of SHBG in PCOS women. Moreover, several randomized clinical trials have shown a significant improvement in ovulation rates in women using the combination of metformin and CC compared to CC alone, although this probably applies more to PCOS women with insulin resistance [5].

The response to CC, used as a treatment for dysovulation in PCOS women, is therefore variable from woman to woman. Since there are no factors that can safely predict the response or not to CC at this time, some authors have suggested a genetic predisposition to explain resistance to treatment. Indeed, CC is metabolized primarily in the liver by cytochrome P450 2D6 (CYP2D6), and to a lesser extent, by cytochromes P450 3A4 and P450 3A5 [56,57,58]. CYP2D6 has a large genetic polymorphism responsible for several different metabolic profiles [59]. The impact of the genetic polymorphism of CYP2D6 on the clinical efficacy of clomiphene citrate is still controversial [56,57,58,59,60,61].

The main limitation of our study is its retrospective nature. The AMH assay was modified in 2016, but we applied a conversion formula for all assays performed after January 2016, thus homogenizing the results and avoiding any measurement bias [35]. Similarly, the ultrasound probe used for the pretherapeutic assessment and, therefore, for the evaluation of the AFC/FNPO was replaced in 2008. Therefore, we decided not to consider AFC/FNPO in the statistical analyses, preferring AMH levels, as previously demonstrated [26,30]. Indeed, the evaluation of AFC/FNPO is operator dependent and can evolve over time with the technical progress of ultrasound probes [28,36], unlike a biological assay such as the AMH assay, which is more reproducible [42,43].

To our knowledge, this study is the largest cohort of anovulatory PCOS patients treated with CC described in the literature and the only one which tries to investigate predictors of resistance to ovulation induction by CC initiated at 50 mg/day. The main strength of our study is, therefore, the large number of patients included. In addition, in this monocentric study, the diagnosis of PCOS and the procedure for monitoring ovulation during CC treatment were standardized. Our results suggest that AMH and SHBG are the only two parameters significantly associated with the risk of resistance to 50 mg/day of CC. However, no cut-off with satisfactory sensitivity and specificity could be established, both for AMH and SHBG, to predict resistance to 50 mg CC.

## Figures and Tables

**Table 1:** Clinical, biological, and ultrasound characteristics of the population (*n* = 283).

Variables			Values *
Age (years)			27.5 ± 3.7
Menstrual Cycles (%)	Regular anovulatory cycles		17 (5.9%)
	Oligomenorrhea		185 (65.5%)
	Amenorrhoea		81 (28.6%)
BMI (kg/m^2^)			25.8 ± 5.4
Waist circumference (cm)			85.2 ± 15.2
Modified Ferriman and Gallwey Score			5.0 (0 to 9.0)
Estradiol (pg/mL)			37.0 (28.0 to 50.0)
FSH (IU/L)			5.0 ± 1.3
LH (IU/L)			5.9 (4.0 to 9.1)
AMH (pmol/L)			71.8 (53.6 to 107.4)
Total testosterone (ng/mL)			0.4 ± 0.2
Δ4-androstenedione (ng/mL)			1.6 (1.2 to 2.2)
17-OHP (ng/mL)			0.6 (0.5 to 0.9)
SHBG (nmol/L)			38.4 (24.0 to 49.8)
SDHEA (μmol/L)			4.6 (3.4 to 6.4)
Fasting insulin (mUI/L)			6.0 (3.1 to 9.8)
Mean ovarian surface (cm^2^)			5.7 ± 1.6
PCOM and/or elevated AMH			280 (99%)
Phenotype PCOS (%)		A = OA + HA + PCOM	225 (79.5%)
		B = OA + HA	3 (1.0%)
		D = OA + PCOM	55 (19.5%)
Mean number of CC cycles			3.2 ± 1.7
Mean number of CC ovulatory cycles			2.3 ± 1.5

* Qualitative variables are expressed as numbers (percentages). * Quantitative variables are expressed as mean ± standard deviation or median (inter-quartile range). Abbreviations: BMI = body mass index; FSH = Follicle Stimulating Hormone; LH = Luteinizing Hormone; AMH = Anti-Müllerian Hormone; 17-OHP = 17-hydroxy-progesterone; SHBG = Sex Hormone Binding Globulin; OA = Oligo or anovulation; HA = Hyperandrogenism; PCOM = Ultrasound polycystic ovaries; CC = Clomiphene Citrate.

**Table 2:** Factors associated with resistance to 50 mg CC in the first cycle of treatment in univariate analyzes.

			Sensitive to 50 mg (*n* = 179)	Resistant to 50 mg (*n* = 104)	*p* Value
Age (years)			27.7 ± 3.8	27.0 ± 3.6	0.12
Menstrual cycles (%)	Regular anovulatory cycles		14 (7.8%)	3 (2.9%)	0.12
	Oligomenorrhea		119 (66.7%)	66 (63.5%)	
	Amenorrhoea		46 (25.5%)	35 (33.6%)	
BMI (kg/m^2^)			24.9 ± 5.2	27.1 ± 5.4	0.001
Waist circumference (cm)			83.4 ± 15.8	88.3 ± 13.7	0.028
Modified Ferriman and Gallwey Score			5.0 (0 to 9.0)	3.0 (0 to 9.0)	0.90
Estradiol (pg/mL)			37.0 (28.0 to 49.5)	39.0 (28.0 to 50.0)	0.87
FSH (IU/L)			5.01 ± 1.4	4.9 ± 1.1	0.48
LH (IU/L)			5.7 (3.9 to 8.9)	6.2 (4.2 to 9.2)	0.33
AMH (pmol/L)			69.4 (51.6 to 101.2)	89.5 (56.0 to 130.0)	0.014
Total testosterone (ng/mL)			0.4 ± 0.2	0.4 ± 0.2	0.046
Δ4-androstenedione (ng/mL)			1.5 (1.1 to 2.1)	1.7 (1.3 to 2.3)	0.023
17-OHP (ng/mL)			0.6 (0.4 to 0.9)	0.7 (0.5 to 0.9)	0.046
SHBG (nmol/L)			39.6 (28.7 to 53.7)	27.2 (18.0 to 40.5)	<0.001
SDHEA (μmol/L)			4.4 (3.0 to 6.4)	4.8 (3.6 to 6.2)	0.43
Fasting insulin (mUI/L)			4.5 (2.9 to 7.1)	7.9 (3.9 to 11.6)	0.002
Mean ovarian surface (cm^2^)			5.7 ± 1.7	5.9 ± 1.5	0.42
PCOS anovulatory phenotypes (%)		A + B	142 (79.3%)	86 (82.7%)	0.48
		D	37 (20.7%)	18 (17.3%)	

Qualitative variables are expressed as numbers (percentages). Quantitative variables are expressed as mean ± standard deviation or median (inter-quartile range). Abbreviations: BMI = body mass index; FSH = Follicle Stimulating Hormone; LH = Luteinizing Hormone; AMH = Anti-Müllerian Hormone; 17-OHP = 17-hydroxy-progesterone; SHBG = Sex Hormone Binding Globulin; OA = Oligo or anovulation; HA = Hyperandrogenism; PCOM = Ultrasound polycystic ovaries; CC = Clomiphene Citrate.

**Table 3:** Independent factors of resistance to 50 mg CC in the first cycle of treatment after multivariate analysis.

Parameters	OR (95% CI)	*p*
AMH (pmol/L)	1.08 (1.03 to 1.14) *	0.002
SHBG (nmol/L)	0.96 (0.94 to 0.99)	0.002

Baseline characteristics associated with a *p* < 0.20 in univariate analyses (Table 2) were implemented into a backward-stepwise multivariable logistic regression model using a removal criterion of *p* > 0.05. Results were expressed using Odds ratios (ORs) as effect sizes with 50 mg non-resistance as reference. * OR expressed for the increase of 10 AMH units. Abbreviations: AMH = Anti-Müllerian Hormone; SHBG = Sex Hormone Binding Globulin.

**Table 4:** Predicted ovulation rates in the first cycle of ovulation induction by CC at 50 mg/day, based on the AMH threshold, the SHBG threshold, and the combined AMH and SHBG thresholds.

		No Ovulation	Ovulation
AMH tested alone	AMH > 86.1 pmol/L	46.12%	53.88%
	AMH < 86.1 pmol/L	29.86%	70.14%
SHBG tested alone	SHBG < 28.3 nmol/L	53,72%	46.28%
	SHBG > 28.3 nmol/L	27.93%	72.07%
AMH and SHBG combined	AMH > 86.1 pmol/L SHBG < 28.3 nmol/L	46.67%	53.33%
	AMH < 86.1 pmol/L SHBG > 28.3 nmol/L	19.42%	80.58%

## Data Availability

The data that support the findings of this study are available from the corresponding author, G.R., upon reasonable request.

## References

[1] Lizneva D., Suturina L., Walker W., Brakta S., Gavrilova-Jordan L., Azziz R. (2016). Criteria, prevalence, and phenotypes of polycystic ovary syndrome. Fertil. Steril..

[2] Jayasena C.N., Franks S. (2014). The management of patients with polycystic ovary syndrome. Nat. Rev. Endocrinol..

[3] The Rotterdam ESHRE/ASRM-Sponsored PCOS Consensus Workshop Group (2004). Revised 2003 consensus on diagnostic criteria and long-term health risks related to polycystic ovary syndrome (PCOS). Hum. Reprod..

[4] The Thessaloniki ESHRE/ASRM-Sponsored PCOS Consensus Workshop Group (2008). Consensus on infertility treatment related to polycystic ovary syndrome. Hum. Reprod..

[5] Balen A.H., Morley L.C., Misso M., Franks S., Legro R.S., Wijeyaratne C.N., Stener-Victorin E., Fauser B.C.J.M., Norman R.J., Teede H. (2016). The management of anovulatory infertility in women with polycystic ovary syndrome: An analysis of the evidence to support the development of global WHO guidance. Hum. Reprod. Update.

[6] Teede H.J., Misso M.L., Costello M.F., Dokras A., Laven J., Moran L., Piltonen T., Norman R.J., International PCOS Network (2018). Recommendations from the international evidence-based guideline for the assessment and management of polycystic ovary syndrome. Hum. Reprod..

[7] Franik S., Le Q.K., Kremer J.A., Kiesel L., Farquhar C. (2022). Aromatase inhibitors (letrozole) for ovulation induction in infertile women with polycystic ovary syndrome. Cochrane Database Syst. Rev..

[8] Hughes E., Collins J., Vandekerckhove P. (1996). Clomiphene citrate for ovulation induction in women with oligo-amenorrhoea. Cochrane Database Syst. Rev.

[9] Homburg R. (2005). Clomiphene citrate—End of an era? A mini-review. Hum. Reprod..

[10] Homburg R. (2008). Polycystic ovary syndrome. Best. Pract. Res. Clin. Obs. Gynaecol..

[11] Beck J.I., Boothroyd C., Proctor M., Farquhar C., Hughes E. (2005). Oral anti-oestrogens and medical adjuncts for subfertility associated with anovulation. Cochrane Database Syst. Rev..

[12] Dewailly D., Hieronimus S., Mirakian P., Hugues J.-N. (2010). Polycystic ovary syndrome (PCOS). Ann. Endocrinol..

[13] Balen A.H. (2013). Ovulation induction in the management of anovulatory polycystic ovary syndrome. Mol. Cell Endocrinol..

[14] Brown J., Farquhar C., Beck J., Boothroyd C., Hughes E. (2009). Clomiphene and anti-oestrogens for ovulation induction in PCOS. Cochrane Database Syst. Rev..

[15] Melo A.S., Ferriani R.A., Navarro P.A. (2015). Treatment of infertility in women with polycystic ovary syndrome: Approach to clinical practice. Clinics.

[16] Wang L., Qi H., Baker P.N., Zhen Q., Zeng Q., Shi R., Tong C., Ge Q. (2017). Altered Circulating Inflammatory Cytokines Are Associated with Anovulatory Polycystic Ovary Syndrome (PCOS) Women Resistant to Clomiphene Citrate Treatment. Med. Sci. Monit..

[17] Ellakwa H.E., Sanad Z.F., Hamza H.A., Emara M.A., Elsayed M.A. (2016). Predictors of patient responses to ovulation induction with clomiphene citrate in patients with polycystic ovary syndrome experiencing infertility. Int. J. Gynecol. Obstet..

[18] Imani B., Eijkemans M.J., te Velde E.R., Habbema J.D., Fauser B.C. (1998). Predictors of patients remaining anovulatory during clomiphene citrate induction of ovulation in normogonadotropic oligoamenorrheic infertility. J. Clin. Endocrinol. Metab..

[19] Imani B., Eijkemans M.J., te Velde E.R., Habbema J.D., Fauser B.C. (1999). Predictors of chances to conceive in ovulatory patients during clomiphene citrate induction of ovulation in normogonadotropic oligoamenorrheic infertility. J. Clin. Endocrinol. Metab..

[20] Overbeek A., Kuijper E.A.M., Hendriks M.L., Blankenstein M.A., Ketel I.J.G., Twisk J.W.R., Hompes P.G.A., Homburg R., Lambalk C.B. (2009). Clomiphene citrate resistance in relation to follicle-stimulating hormone receptor Ser680Ser-polymorphism in polycystic ovary syndrome. Hum. Reprod..

[21] Xi W., Yang Y., Mao H., Zhao X., Liu M., Fu S. (2016). Circulating anti-mullerian hormone as predictor of ovarian response to clomiphene citrate in women with polycystic ovary syndrome. J. Ovarian Res..

[22] Mercorio A., Della Corte L., De Angelis M.C., Buonfantino C., Ronsini C., Bifulco G., Giampaolino P. (2022). Ovarian Drilling: Back to the Future. Medicina.

[23] Practice Committees of the American Society for Reproductive Medicine and the Society for Reproductive Endocrinology and Infertility (2021). Diagnosis and treatment of luteal phase deficiency: A committee opinion. Fertil. Steril..

[24] Escobar-Morreale H.F., Carmina E., Dewailly D., Gambineri A., Kelestimur F., Moghetti P., Pugeat M., Qiao J., Wijeyaratne C.N., Witchel S.F. (2012). Epidemiology, diagnosis and management of hirsutism: A consensus statement by the Androgen Excess and Polycystic Ovary Syndrome Society. Hum. Reprod. Update.

[25] Jonard S., Robert Y., Dewailly D. (2005). Revisiting the ovarian volume as a diagnostic criterion for polycystic ovaries. Hum. Reprod..

[26] Dewailly D., Gronier H., Poncelet E., Robin G., Leroy M., Pigny P., Duhamel A., Catteau-Jonard S. (2011). Diagnosis of polycystic ovary syndrome (PCOS): Revisiting the threshold values of follicle count on ultrasound and of the serum AMH level for the definition of polycystic ovaries. Hum. Reprod..

[27] Balen A.H., Laven J.S.E., Tan S.-L., Dewailly D. (2003). Ultrasound assessment of the polycystic ovary: International consensus definitions. Hum. Reprod. Update.

[28] Dewailly D., Lujan M.E., Carmina E., Cedars M.I., Laven J., Norman R.J., Escobar-Morreale H.F. (2014). Definition and significance of polycystic ovarian morphology: A task force report from the Androgen Excess and Polycystic Ovary Syndrome Society. Hum. Reprod. Update.

[29] Robin G., Gallo C., Catteau-Jonard S., Lefebvre-Maunoury C., Pigny P., Duhamel A., Dewailly D. (2012). Polycystic Ovary-Like Abnormalities (PCO-L) in women with functional hypothalamic amenorrhea. J. Clin. Endocrinol. Metab..

[30] Fraissinet A., Robin G., Pigny P., Lefebvre T., Catteau-Jonard S., Dewailly D. (2017). Use of the serum anti-Müllerian hormone assay as a surrogate for polycystic ovarian morphology: Impact on diagnosis and phenotypic classification of polycystic ovary syndrome. Hum. Reprod..

[31] Jonard S., Robert Y., Cortet-Rudelli C., Pigny P., Decanter C., Dewailly D. (2003). Ultrasound examination of polycystic ovaries: Is it worth counting the follicles?. Hum. Reprod..

[32] Dewailly D., Pigny P., Soudan B., Catteau-Jonard S., Decanter C., Poncelet E., Duhamel A. (2010). Reconciling the definitions of polycystic ovary syndrome: The ovarian follicle number and serum anti-Müllerian hormone concentrations aggregate with the markers of hyperandrogenism. J. Clin. Endocrinol. Metab..

[33] Pigny P., Jonard S., Robert Y., Dewailly D. (2006). Serum anti-Mullerian hormone as a surrogate for antral follicle count for definition of the polycystic ovary syndrome. J. Clin. Endocrinol. Metab..

[34] Pigny P., Merlen E., Robert Y., Cortet-Rudelli C., Decanter C., Jonard S., Dewailly D. (2003). Elevated serum level of anti-mullerian hormone in patients with polycystic ovary syndrome: Relationship to the ovarian follicle excess and to the follicular arrest. J. Clin. Endocrinol. Metab..

[35] Pigny P., Gorisse E., Ghulam A., Robin G., Catteau-Jonard S., Duhamel A., Dewailly D. (2016). Comparative assessment of five serum antimüllerian hormone assays for the diagnosis of polycystic ovary syndrome. Fertil. Steril..

[36] Coelho Neto M.A., Ludwin A., Borrell A., Benacerraf B., Dewailly D., da Silva Costa F., Condous G., Alcazar J.L., Jokubkiene L., Guerriero S. (2018). Counting ovarian antral follicles by ultrasound: A practical guide. Ultrasound Obstet. Gynecol..

[37] Gadalla M.A., Huang S., Wang R., Norman R.J., Abdullah S.A., El Saman A.M., Ismail A.M., van Wely M., Mol B.W.J. (2018). Effect of clomiphene citrate on endometrial thickness, ovulation, pregnancy and live birth in anovulatory women: Systematic review and meta-analysis. Ultrasound Obstet. Gynecol..

[38] Harrell F.E., Lee K.L., Mark D.B. (1996). Multivariable prognostic models: Issues in developing models, evaluating assumptions and adequacy, and measuring and reducing errors. Stat. Med..

[39] Buuren S.v., Groothuis-Oudshoorn K. (2011). Mice: Multivariate Imputation by Chained Equations in *R*. J. Stat. Softw..

[40] Segalas C., Leyrat C., Carpenter J.R., Williamson E. (2023). Propensity score matching after multiple imputation when a confounder has missing data. Stat. Med..

[41] Mahran A., Abdelmeged A., El-Adawy A.R., Eissa M.K., Shaw R.W., Amer S.A. (2013). The predictive value of circulating anti-Müllerian hormone in women with polycystic ovarian syndrome receiving clomiphene citrate: A prospective observational study. J. Clin. Endocrinol. Metab..

[42] Dewailly D., Andersen C.Y., Balen A., Broekmans F., Dilaver N., Fanchin R., Griesinger G., Kelsey T.W., La Marca A., Lambalk C. (2014). The physiology and clinical utility of anti-Mullerian hormone in women. Hum. Reprod. Update.

[43] Dumont A., Robin G., Catteau-Jonard S., Dewailly D. (2015). Role of Anti-Müllerian Hormone in pathophysiology, diagnosis and treatment of Polycystic Ovary Syndrome: A review. Reprod. Biol. Endocrinol..

[44] Pellatt L., Rice S., Dilaver N., Heshri A., Galea R., Brincat M., Brown K., Simpson E.R., Mason H.D. (2011). Anti-Müllerian hormone reduces follicle sensitivity to follicle-stimulating hormone in human granulosa cells. Fertil. Steril..

[45] Köninger A., Sauter L., Edimiris P., Kasimir-Bauer S., Kimmig R., Strowitzki T., Schmidt B. (2014). Predictive markers for the FSH sensitivity of women with polycystic ovarian syndrome. Hum. Reprod..

[46] Amer S.A., Li T.C., Ledger W.L. (2009). The value of measuring anti-Mullerian hormone in women with anovulatory polycystic ovary syndrome undergoing laparoscopic ovarian diathermy. Hum. Reprod..

[47] Gülşen M.S., Ulu İ., Köpük Y.Ş., Kıran G. (2019). The role of anti-Müllerian hormone in predicting clomiphene citrate resistance in women with polycystic ovarian syndrome. Gynecol. Endocrinol..

[48] Hestiantoro A., Negoro Y.S., Afrita Y., Wiweko B., Sumapradja K., Natadisastra M. (2016). Anti-Müllerian hormone as a predictor of polycystic ovary syndrome treated with clomiphene citrate. Clin. Exp. Reprod. Med..

[49] Vaiarelli A., Drakopoulos P., Blockeel C., De Vos M., van de Vijver A., Camus M., Cosyns S., Tournaye H., Polyzos N.P. (2016). Limited ability of circulating anti-Müllerian hormone to predict dominant follicular recruitment in PCOS women treated with clomiphene citrate: A comparison of two different assays. Gynecol. Endocrinol..

[50] Giampaolino P., Della Corte L., De Rosa N., Mercorio A., Bruzzese D., Bifulco G. (2018). Ovarian volume and PCOS: A controversial issue. Gynecol. Endocrinol..

[51] Zhu J.-L., Chen Z., Feng W.-J., Long S.-L., Mo Z.-C. (2019). Sex hormone-binding globulin and polycystic ovary syndrome. Clin. Chim. Acta.

[52] Calzada M., López N., Noguera J.A., Mendiola J., Hernández A.I., Corbalán S., Sanchez M., Torres A.M. (2019). AMH in combination with SHBG for the diagnosis of polycystic ovary syndrome. J. Obstet. Gynaecol..

[53] Simó R., Sáez-López C., Barbosa-Desongles A., Hernández C., Selva D.M. (2015). Novel insights in SHBG regulation and clinical implications. Trends Endocrinol. Metab..

[54] Deswal R., Yadav A., Dang A.S. (2018). Sex hormone binding globulin—An important biomarker for predicting PCOS risk: A systematic review and meta-analysis. Syst. Biol. Reprod. Med..

[55] Sachdeva G., Gainder S., Suri V., Sachdeva N., Chopra S. (2019). Comparison of Clinical, Metabolic, Hormonal, and Ultrasound Parameters among the Clomiphene Citrate-Resistant and Clomiphene Citrate-Sensitive Polycystic Ovary Syndrome Women. J. Hum. Reprod. Sci..

[56] Ghobadi C., Amer S., Lashen H., Lennard M.S., Ledger W.L., Rostami-Hodjegan A. (2009). Evaluation of the relationship between plasma concentrations of en- and zuclomiphene and induction of ovulation in anovulatory women being treated with clomiphene citrate. Fertil. Steril..

[57] Mürdter T.E., Kerb R., Turpeinen M., Schroth W., Ganchev B., Böhmer G.M., Igel S., Schaeffeler E., Zanger U., Brauch H. (2012). Genetic polymorphism of cytochrome P450 2D6 determines oestrogen receptor activity of the major infertility drug clomiphene via its active metabolites. Hum. Mol. Genet..

[58] Ghobadi C., Gregory A., Crewe H.K., Rostami-Hodjegan A., Lennard M.S. (2008). CYP2D6 is primarily responsible for the metabolism of clomiphene. Drug Metab. Pharmacokinet..

[59] Zhou S.-F. (2009). Polymorphism of human cytochrome P450 2D6 and its clinical significance: Part II. Clin. Pharmacokinet..

[60] Robin C., Hennart B., Broly F., Gruchala P., Robin G., Catteau-Jonard S. (2021). Could Cytochrome P450 2D6, 3A4 and 3A5 Polymorphisms Explain the Variability in Clinical Response to Clomiphene Citrate of Anovulatory PCOS Women?. Front. Endocrinol..

[61] Ji M., Kim K.-R., Lee W., Choe W., Chun S., Min W.-K. (2016). Genetic Polymorphism of CYP2D6 and Clomiphene Concentrations in Infertile Patients with Ovulatory Dysfunction Treated with Clomiphene Citrate. J. Korean Med. Sci..

